# Socioeconomic disparities in organized sports participation and physical activity among a population based sample of preschool children: a cross-sectional study

**DOI:** 10.1186/s12887-025-05651-3

**Published:** 2025-04-16

**Authors:** Charlotte Wilén, Viktor H. Ahlqvist, Chu Chen, Martin Neovius, Cecilia Magnusson, Pontus Henriksson, Micael Dahlén, Erik Sander, Daniel Berglind

**Affiliations:** 1 Department of Global Public Health, Karolinska Institutet, Stockholm, Sweden; 2 Centre for Epidemiology and Community Medicine, Stockholm County Council, Stockholm, Sweden; 3 Department of Medicine, Unit, Karolinska Institutet, Clinical Epidemiology Division, Solna, Sweden; 4 Department of Health, Medicine and Caring Sciences, Linköping University, Linköping, Sweden; 5 Center for Wellbeing, Welfare and Happiness, Stockholm School of Economics, Stockholm, Sweden

**Keywords:** Physical activity, Organized sports, Socioeconomic status, Children, Preschool, Accelerometer

## Abstract

**Background:**

Encouraging children to participate in organized sports could increase physical activity and may promote lifelong healthy habits. There are socioeconomic disparities in organized sports participation in school-aged children and adolescents. However, it is not known if these socioeconomic disparities exist among preschool-aged children.

**Objective:**

The purpose of this study was to examine (i) the association between organized sports participation and physical activity (ii) differences in organized sports participation according to socioeconomic status among preschool children.

**Methods:**

The study sample was a representative population-based sample of 2935 preschool-aged children (48.3% girls; age range 2–6 years) in Stockholm, Sweden. Physical activity was measured using GT3X + accelerometers for one week at the fall of 2020 or in the spring of 2021 and organized sports participation was parent-reported. Parental education and a Stockholm based socioeconomic index were used to examine socioeconomic disparities. Linear regression was used to estimate associations between organized sports participation and physical activity and multinomial logistic regression was used to estimate the differences in organized sports participation over parental education and neighborhood socioeconomic index.

**Results:**

The study population had a mean age of 4.5 years (SD = 0.9), consisted of 48.3% girls and spent in average 46.5 min (SD = 15.4) engaged in moderate to vigorous physical activity per day. Additionally, 1,658 children (56.5%) did not participate in organized sports. Participation in organized sports once a week or more was associated with a 2.8-min increase in average daily moderate to vigorous physical activity (95% CI; 1.56, 4.06), compared to not participating. Further, both living in a higher socioeconomic index area and higher parental education was associated with higher organized sports participation.

**Conclusions:**

Participation in organized sports indicates a modest contribution to physical activity among preschool-aged children. However, participation in organized sports varies according to neighborhood socioeconomic index and parental education. These findings highlight the importance of targeting organized sport participation according to socioeconomic gradients, to moderate inequities in access and opportunity to organized sport.

**Supplementary Information:**

The online version contains supplementary material available at 10.1186/s12887-025-05651-3.

## Key points

• This study found that participation in organized sports is associated with a modestly higher level of physical activity among preschool-aged children living in Stockholm.  However, 56.5% of the children did not participate in any organized sports.

• Further, we found that participation in organized sports differs depending on socioeconomic status, highlighting the importance of targeted interventions to reduce these differences.

**Supplementary Information:**

The online version contains supplementary material available at 10.1186/s12887-025-05651-3.

## Introduction

Physical activity is a crucial component of healthy early childhood development, playing a pivotal role in promoting physical, cognitive, and social skills among preschool-aged children [1]. Regular engagement in physical activities not only fosters motor skills and physical fitness [2] but also establishes the foundation for a lifelong appreciation of an active lifestyle [3]. While the importance of physical activity during early childhood is well-established [1], disparities in physical activity levels have been observed among children of varying socioeconomic backgrounds [4].

Swedish preschool-aged children’s physical activity levels are low, especially outside preschool hours and on weekend days [5] and few meet the current physical activity guidelines of at least 60 min of daily moderate to vigorous physical activity (MVPA) [6]. One potential method to increase physical activity in children is to encourage their participation in organized sports which can provide structured and supervised opportunities for physical activity [7], social interaction, improved mental health [8], improved bone health [9] and decreased risk for cardiovascular disease [10]. Moreover, childhood exposure to organized sports may be influential on physical activity levels later in life [11].

We have previously showed a positive association between participation in organized sports and objectively measured MVPA in a socioeconomic homogenous sample of preschool-aged children [12]. There is evidence of socioeconomic disparities in organized sport participation among children and adolescents [13]. However, it is not known if these socioeconomic disparities exist among preschool-aged children. Children from socioeconomically advantaged backgrounds might have greater access to organized sports facilities, qualified coaching, and parental encouragement, potentially leading to higher levels of physical activity. Conversely, children from socioeconomically disadvantaged backgrounds may face barriers such as limited financial resources, reduced access to recreational facilities, and increased time constraints, which could impact their involvement in organized sports and overall physical activity levels [13].

Understanding the relationship between organized sports participation and physical activity across the socioeconomic gradient in preschool-aged children could inform the development of targeted interventions and policies aimed at reducing physical activity disparities and promoting equitable opportunities for active engagement during early childhood. Moreover, studies examining the association between organized sports participation and physical activity are often limited in size and rarely focus on preschool-aged children. Thus, the aim of this study was to examine (i) the association between organized sports participation and accelerometer-measured physical activity and (ii) examine differences in organized sports participation according to parental education and neighborhood socioeconomic index using a representative population-based sample of preschool-aged children in Stockholm, Sweden.

## Methods

### Study design and setting

The current study is cross-sectional and based on data collected for the Children’s Physical Activity by Policy (CAP) trial. Further details of the sampling procedures have been published elsewhere [14]. In brief, preschools were invited in a random order from each of the 11 districts of Region Stockholm until the number of preschools agreeing to participate reached approximately 30% of all public preschools in that district, resulting in a total of 124 preschools. Data were collected over a one-week period during the fall of 2020 and followed up with another one-week period in the spring of 2021. For this study, we primarily retained data recorded during the fall measure. However, in instances where the child was absent or did not participate during the fall measure, we retained the spring measure.

### Derivation of study population

**Fig. 1:**
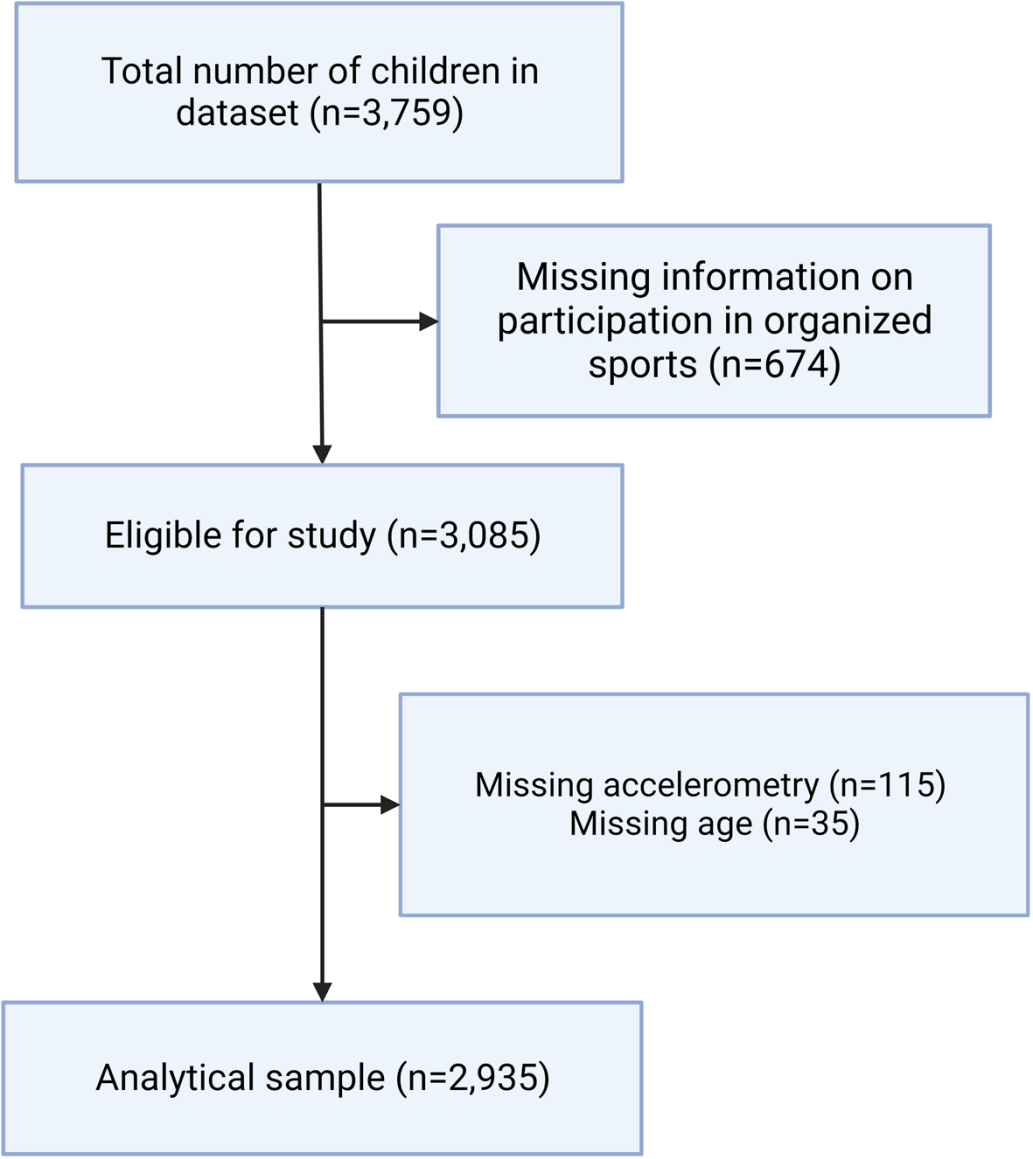
Flowchart of the derivation of the analytical sample

Out of all 3,759 children participating in the CAP trial, 2,935 children were included in the current study (Fig. 1). First, 674 children were excluded due to no information on organized sports participation. Further, 115 children were excluded because of missing accelerometry data and another 35 children due to no information on age. For a few children (*N* = 114), weight and height were not available at the same time point as the accelerometer data, so we complemented it from the other time point and derived a technical variable to adjust for the season of weight/height recording.

### Organized sports

Organized sport is defined as *“physical activity that is directed by adult or youth leaders and involves rules and formal practice and competition*” [15]. To assess the number of times children participated in organized sports, parents received a questionnaire daily during the measurement week in fall and in spring. The questionnaire contained questions about if the child participates in organized sports (yes/no) and if so, the time and duration and type of sport (free text). However, these details—time, duration, and type—were not taken into account in the analysis. If the parents answered that the child participated in organized sports one day, that was considered as one occasion. Furthermore, if the child participated in more than one sport per day, each sport was treated as a distinct occasion. The number of occasions in organized sports is calculated as a mean of occasions per week (both seasons included).

### Physical activity and sedentary time

The measurements of physical activity taken into consideration in this study are MVPA, light physical activity (LPA), sedentary time (ST) and steps. These measurements are collected through triaxial GT3X + accelerometers which the children were instructed to wear on their non-dominant wrist 24 h a day, except during water activities. The classification of physical activity and ST followed the cut-offs for children described by Hildebrand et al. [16, 17], while steps were measured using the Verisense step algorithm [18]. Accelerometer data was considered invalid if a child wore the device for less than 10 h on a given day or had fewer than 3 valid days of wear time. Furthermore, the fraction of children reaching The World Health Organization’s (WHO) recommendations for preschool children, of at least 60 min of MVPA each day [6] was assessed.

### Neighborhood socioeconomic index and highest parental education

The neighborhood socioeconomic index is an area-based index, based on data from 2019, used in Region Stockholm to examine socioeconomic conditions [19]. In brief, the index is the first principal component derived from a 10-level principal component analysis of the area-level proportions of unemployment, financial assistance, pre-secondary education, single parents, foreign-born residents, median income, and average living space. Each child is assigned the neighborhood socioeconomic index corresponding to the area where their preschool is located. To adjust for and stratify based on this index, quintiles of the index distributions were calculated.

The highest parental educational level was collected through questionnaires and divided into three categories: (i) secondary school (compulsory), (ii) upper secondary school and (iii) university. Due to missing information on highest parental education (*N* = 293), imputation was performed by creating a dummy variable, which was addressed for in sensitivity analysis.

### Covariates

Sex and age were derived from the children’s personal registry number that was reported by their parents at baseline when providing consent to participate in the CAP study. Age was rounded to the closest 0.5 years and half a year has been added in the spring measure. Weight and height were measured with validated scales and stadiometers and were used to calculate body mass index. For a few children (*N* = 114), weight and height were not available at the same time point as the accelerometer data, so we complemented it from the other time point and derived a technical variable to adjust for the season of weight/height recording. Furthermore, body mass index was used to estimate obesity status that was categorized into three categories: (i) normal weight, (ii) overweight and (iii) obesity, based on the age- and sex-specific criteria developed by Cole et al. [20].

### Statistical methods

To estimate the mean differences in physical activity between those participating in organized sports, on average either once a week or more, and those who did not participate in organized sports, we employed linear regression. The results are provided as crude and adjusted for age, sex, accelerometer wear time, obesity status, number of days participating in the questionnaire on organized sports, highest parental education, and neighborhood socioeconomic index. Furthermore, except for in sensitivity analysis, we adjust for the fact that accelerometer data is sometimes taken from fall 2020 (*N* = 2,511) and sometimes from spring 2021 (*N* = 310), as well as the fact that information on obesity status and accelerometer data do not originate from the same season in some cases. To estimate the differences in organized sports participation over neighborhood socioeconomic index and parental education, we employed a multinomial logistic regression and post-estimate the probabilities of participating in organized sport (prevalence’s, henceforth). This analysis is not adjusted for any covariates since it aims to highlight public health needs, rather than the counterfactual scenario had they experienced the same covariate distribution in every socioeconomic group. All analyses were conducted using clustered standard errors at the preschool level to account for the correlation among children in the same preschool. Statistical analyses were conducted using Stata 17.0.

### Sensitivity analysis

The linear regression used to estimate the mean differences in physical activity between children who participated in organized sports and those who did not, was repeated without the children (*N* = 114) where information on obesity status and accelerometer data did not originate from the same season. Furthermore, the analysis was repeated after excluding children with accelerometer data from spring (*N* = 310). Since the children were asked not to wear the accelerometer during water activities, i.e., physical activity generated during swimming will not be accounted for, a third sensitivity analysis were constructed when excluding children who participated in swimming (*N* = 185). A fourth sensitivity analysis was performed after excluding the children with no information on parental education (*N* = 291). Lastly, some children extended accelerometer use beyond the standard two-week period, thereby capturing participation in organized sports for an extended duration, while others had limited data covering only a few days. A sensitivity analysis, involving the exclusion of children with fewer than 3 or more than 16 days of registered participation in organized sports, regardless of their responses, was conducted (*N* = 114). We adjusted for the same covariates as in the main analysis, except for the two first sensitivity analyses where we did not include the variable to adjust for the season of weight/height recording.

## Results

### Descriptive characteristics

Out of the 2,935 children (48.3% girls; mean age 4.5 (SD = 0.9), ranging between 2–6 years), 56.5% did not participate in organized sports, while 26.1% of the children participated up to once a week on average, and 17.4% of the children participated more than once a week on average (Table 1). The four most reported organized sports were (i) swimming (6.3%), (ii) gymnastics (5.7%), (iii) dance/song/theatre (5.5%) and (iv) football (5.3%).

**Table 1:** Descriptive characteristics of participating children in total and across participation in organized sports

Number of times participating in organized sports per week				
	Total	0	> 0–1	1 +
	*N* = 2,935	*N* = 1,658	*N* = 765	*N* = 512
**Girls, N (%)**	1,417 (48.3%)	736 (44.4%)	400 (52.3%)	281 (54.9%)
**Age, Mean (SD)**	4.5 (0.9)	4.3 (0.9)	4.7 (0.8)	4.8 (0.8)
**Obesity status, N (%)**				
Normal	2,356 (80.3%)	1,319 (79.6%)	630 (82.4%)	407 (79.5%)
Overweight	487 (16.6%)	283 (17.1%)	116 (15.2%)	88 (17.2%)
Obese	92 (3.1%)	56 (3.4%)	19 (2.5%)	17 (3.3%)
**Highest parental education, N (%)**				
Secondary school	37 (1.3%)	25 (1.5%)	4 (0.5%)	8 (1.6%)
Upper secondary school	365 (12.4%)	228 (13.8%)	78 (10.2%)	59 (11.5%)
University level	2,242 (76.4%)	1,212 (73.1%)	641 (83.8%)	389 (76.0%)
No information	291 (9.9%)	193 (11.6%)	42 (5.5%)	56 (10.9%)
**Steps (count), Mean (SD)**	8162.8 (1523.7)	7986.0 (1506.2)	8419.3 (1544.0)	8351.9 (1474.4)
**Sedentary time (min), Mean (SD)**	450.4 (60.0)	454.4 (61.1)	446.3 (56.5)	443.9 (60.4)
**Light physical activity (min), Mean (SD)**	220.9 (43.0)	218.8 (45.1)	224.8 (40.2)	222.2 (39.5)
**Moderate to vigorous physical activity (min), Mean (SD)**	46.5 (15.4)	44.6 (15.2)	48.8 (15.3)	49.3 (15.4)
**Meets MVPA recommendations, ≥ 60 min/day, N (%)**	502 (17.1%)	243 (14.7%)	155 (20.3%)	104 (20.3%)
**Wear-time (min), Mean (SD)**	720.4 (68.3)	715.4 (71.0)	730.6 (60.6)	721.3 (68.6)
**Neighborhood socioeconomic index, N (%)**				
Very low (Q1)	599 (20.4%)	386 (23.3%)	105 (13.7%)	108 (21.1%)
Low (Q2)	729 (24.8%)	440 (26.5%)	200 (26.1%)	89 (17.4%)
Medium (Q3)	500 (17.0%)	273 (16.5%)	157 (20.5%)	70 (13.7%)
High (Q4)	576 (19.6%)	321 (19.4%)	148 (19.3%)	107 (20.9%)
Very high (Q5)	531 (18.1%)	238 (14.4%)	155 (20.3%)	138 (27.0%)
**The four most common organized sports, N (%)**				
Swimming	185 (6.3%)	NA	80 (10.5%)	105 (20.5%)
Gymnastics	168 (5.7%)	NA	80 (10.5%)	88 (17.2%)
Dance, song, theater	161 (5.5%)	NA	72 (9.4%)	89 (17.4%)
Football	157 (5.3%)	NA	69 (9.0%)	88 (17.2%)

Those who did not participate in organized sports were more likely to be boys (55.6% vs 42.1%), younger (4.3 vs 4.8 mean years of age), and wear the accelerometer for a shorter period (715.4 vs 721.3 min per day), as compared to children participating once or more a week (Table 1).

### Association between participating in organized sports and different levels of physical activity

Children who participated in organized sports were more physically active (Table 2). After adjusting for covariate differences, participation in organized sports once a week was associated with a 2.8-min increase in average daily MVPA (95% CI: 1.52,4.02, *P*-value < 0.001) and 40% increased odds of reaching WHO’s recommendations of daily MVPA (odds ratio 1.40; 95% CI: 1.13,1.78, *P*-value = 0.003), compared to the children that never participated in organized sports. Children who participated in organized sports once a week also engaged 5.3 min/day less in ST, compared to children not participating in organized sports (95% CI: − 10.37,− 0.27, *P*-value 0.039).

**Table 2:** Mean daily minutes, mean differences in moderate to vigorous physical activity, light physical activity, and sedentary time according to participation in organized sports, as well as the percentage and odds ratios of reaching MVPA recommendations (≥ 60 min per day)

**Sedentary time (min)**									
		**Crude analysis**				Adjusted analysis*			
	**Observations No., (%)**	**Mean**	**Mean difference**	**95% Cl**	**P-value**	**Mean**	**Mean difference**	**95% Cl**	***P*-value**
** Per occasion organized sport**	2,935 (100%)		− 3.0	− 6.12,0.20	0.066		− 3.1	− 5.96,− 0.28	0.031
** Average number of occasions**									
** 0**	1,658 (56.5%)	454.4	ref	-		453.5	ref	-	
** > 0–1**	765 (26.1%)	446.3	− 8.1	− 13.24,− 2.94	0.002	448.2	− 5.3	− 10.37,− 0.27	0.039
** 1 + **	512 (17.4%)	443.9	− 10.5	− 17.59,− 3.43	0.004	443.7	− 9.8	− 16.42,− 3.18	0.004
**Light physical activity (min)**									
		**Crude analysis**				Adjusted analysis*			
	**Observations No., (%)**	**Mean**	**Mean difference**	**95% Cl**	**P-value**	**Mean**	**Mean difference**	**95% Cl**	***P*-value**
** Per occasion organized sport**	2,935 (100%)		0.8	− 1.33,2.99	0.45		− 0.4	− 2.38,1.59	0.694
** Average number of occasions**									
** 0**	1,658 (56.5%)	218.8	ref	-		220.5	ref	-	
** > 0–1**	765 (26.1%)	224.8	6.0	2.17,9.85	0.002	222.1	1.6	− 2.21,5.32	0.417
** 1 + **	512 (17.4%)	222.2	3.4	− 1.28,8.03	0.155	220.6	0.1	− 4.42,4.69	0.953
**Moderate to vigorous physical activity (min)**									
		**Crude analysis**				Adjusted analysis*			
	**Observations No., (%)**	**Mean**	**Mean difference**	**95% Cl**	**P-value**	**Mean**	**Mean difference**	**95% Cl**	***P*-value**
** Per occasion organized sport**	2,935 (100%)		2.0	1.14,2.83	< 0.001		1.1	0.41,1.79	0.002
** Average number of occasions**									
** 0**	1,658 (56.5%)	44.6	ref	-		45.3	ref	-	
** > 0–1**	765 (26.1%)	48.8	4.2	2.89,5.43	< 0.001	48.1	2.8	1.52,4.02	< 0.001
** 1 + **	512 (17.4%)	49.3	4.7	3.14,6.21	< 0.001	48.1	2.8	1.56,4.06	< 0.001
**Meets recommendations, ≥ 60 min MVPA/day**									
		**Crude analysis**				Adjusted analysis*			
	**Observations No., (%)**	**% Reaching recommendations**	**Odds ratio**	**95% Cl**	**P-value**	**% Reaching recommendations**	**Odds ratio**	**95% Cl**	***P*-value**
** Per occasion organized sport**	2,935 (100%)		1.2	1.07,1.33	0.002		1.1	0.99,1.25	0.062
** Average number of occasions**									
** 0**	1,658 (56.5%)	14.7	ref	-		15.3	ref	-	
** > 0–1**	765 (26.1%)	20.3	1.5	1.19,1.83	< 0.001	19.8	1.4	1.13,1.78	0.003
** 1 + **	512 (17.4%)	20.3	1.5	1.14,1.94	0.004	18.6	1.3	1.00,1.70	0.052

*Adjusted for age, sex, highest parental education, accelerometer wear time, obesity status, number of daily reports of organized sports, neighborhood socioeconomic index, the season in which information on accelerometer data is taken and season of weight/length recording

### Association between socioeconomic factors and organized sports

**Fig. 2:**
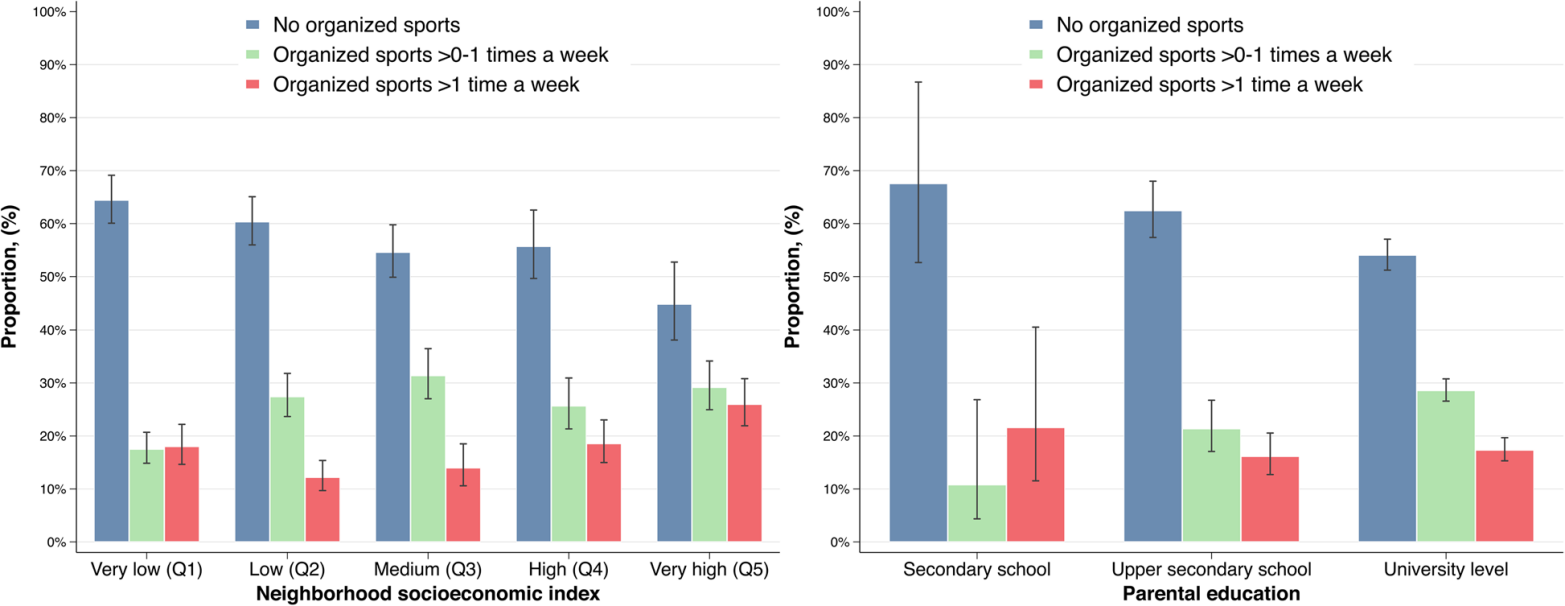
Prevalence of children participating in organized sports, stratified on neighborhood socioeconomic index and parental education. 291 children were excluded in graph concerning highest parental education due to missing information

Children’s participation in organized sports varied markedly by neighborhood socioeconomic index and parental education (Fig. 2, Supplementary Table 1). A majority (64%; 95% CI: 60–69%) of children in the lowest quintile of the neighborhood socioeconomic index never participated in organized sports, whereas 45% (95% CI: 38–53%) of children in the highest quintile of the neighborhood socioeconomic index abstained from participating in organized sports.

Children with low parental education were also less likely to participate in organized sports, with a non-participation rate of 68% (95% CI: 53–87%), compared to 54% (95% CI: 51–57%) among children with higher parental education. The gradient of non-participation across both neighborhood socioeconomic index and parental education followed a virtually linear trend. This trend appears to be primarily driven by whether the child participated at all, as the participation in organized sports more than once a week on average did not vary across either neighborhood socioeconomic index or parental education.

### Sensitivity analysis

There were no distinct differences in the sensitivity analyses compared to the main analysis (Supplementary Table 2). Specifically, excluding the children with non-matching seasonal measures on certain variables (N excluded = 114), retaining only those with the fall measure (N excluded = 310), excluding the children who participated in swimming (N excluded = 185), excluding children with missing parental education (N excluded = 291) and remaining only children where the number of days of registered participation in organized sports fell within the range of 3 to 16 days (N excluded = 114) did not markedly alter the estimates as compared to the main analysis.

## Discussion

In this study, we investigated the associations between organized sports participation and physical activity levels while exploring socioeconomic disparities among a population-based sample of preschool-aged children in Stockholm. Our findings showed that approximately 43% of children participated in organized sports. Children who participated in organized sports spent on average 2.8 min (5.6%) more in daily MVPA and had 40% higher odds of reaching WHO’s physical activity recommendations. Furthermore, socioeconomic status may have influenced participation in organized sports, with children from the lowest neighborhood socioeconomic index group (non-participation: 64%, 95% CI: 60–69%) and those with low parental education (non-participation: 68%, 95% CI: 53–87%) being less likely to participate compared to their counterparts from the highest socioeconomic group (45%, 95% CI: 38–53%) and highest parental education group (54%, 95% CI: 51–57%).

### Comparison with previous research

Previous research showing an association between organized sports participation and physical activity is mostly limited to school-aged children and adolescents [13, 15, 21]. We have previously showed that compared to children not participating in organized sports, organized sports participation was associated with 6.0 min (10%) more MVPA per day in a sample of preschool-aged children living with highly educated parents (81% with a university education) in a neighborhood with a high socioeconomic index [12]. That is almost twice as much MVPA associated with organized sports participation as shown in this study. However, differences between the two study populations may explain the discrepancies in results. First (i), the current study included several areas with lower socioeconomic index and parents with on average lower levels of education (75% with a university education). Second (ii), in the current study fewer children participated in organized sports (43% vs. 50%). Such population sample characteristics may to some extent explain the observed differences in MVPA associated with organized sports participation between the two studies.

Consistent with previous meta-analysis on socioeconomic disparities in organized sports participation [13], this study indicated that children’s participation in organized sports varied by socioeconomic status. Both higher neighborhood socioeconomic index and parental education displayed a linear association with children’s organized sports participation once a week. However, these socioeconomic disparities did not exist for participation in organized sports more than once a week. This finding contradicts previous research [15] and needs further investigation. We speculate, however, that families already engaged in organized sports (e.g., multiple times a week) participate regardless of socioeconomic status. Specifically, starting participation may pose a greater challenge than increasing it, likely due to barriers such as lack of motivation and routine. Conversely, those considering participation once a week may face more pronounced barriers compared to those looking to increase their frequency.

### Implication of findings

Our findings provide valuable insights into the role of organized sports in promoting physical activity during the crucial early childhood years, while also shedding light on socioeconomic disparities in the access to organized sports participation.

Organized sports participation provides children with structured physical activity but can also provide mental health benefits, foster social interactions and promote active lifestyles from an early age [7, 8, 11, 22].

This study found that participation in organized sports was associated with a 2.8-min increase in MVPA and a 5.3-min decrease in ST compared to non-participation—a modest but positive result. While the magnitude may seem small, they could mark the start of a positive physical activity trajectory, considering that establishing healthy habits early often persists into adulthood [3]. However, taken at face value, the short-term relationship between organized sports on children’s physical activity in this study remains modest. As such, the identified socioeconomic discrepancies raise important concerns about the potential perpetuation of health inequalities among preschool-aged children. Several factors could contribute to the observed socioeconomic discrepancies. Financial constraints might restrict the ability of lower-income families to enroll their children in organized sports programs that often involve registration fees, equipment costs, and transportation expenses. Additionally, cultural and social norms within certain communities/areas could influence parental attitudes towards sports participation, potentially limiting opportunities for physical activity among preschool-aged children [13]. Limited access to organized sports programs in economically disadvantaged communities may hinder opportunities for physical activity and, subsequently, impact the overall health and well-being of children in these areas [9]. Addressing this disparity is crucial for promoting health equity and ensuring that all children, regardless of socioeconomic background, have equal opportunities to engage in physical activities that are vital for their development.

To mitigate the socioeconomic disparities in organized sports participation and physical activity, a system-based approach and policies are warranted [23]. Governments and community organizations should focus on creating affordable and accessible sports programs in underserved neighborhoods, offering subventions to support children from lower-income families. Indeed, financial incentive programs have shown to effectively increase children’s organized sports participation [24].

### Strengths and limitations

There are several strengths of this study: first (ii) the study population was randomly selected population-based sample including approximately 10% of the preschool population in Stockholm. However, since the study only includes participants from Stockholm, the results may be primarily generalizable to larger urban cities with comparable settings. Second, physical activity was measured by accelerometers, which is the preferred method for population physical activity surveillance [25]. Third, we used both detailed data on Stockholm city socioeconomic index areas and parental education level to investigate socioeconomic disparities in organized sports participation and physical activity.

While our study provides valuable insights into the associations between organized sports participation and physical activity among preschool-aged children, it is essential to acknowledge its limitations. First, the observational and cross-sectional nature of the study preclude any inference about causality, and we cannot rule out that our results may be explained by unmeasured confounders or reverse causality, i.e., children who are more active chose to participate in organized sports and not vice versa. Second, the limitations of the data collection methods should be considered. Organized sport is recorded per occasion, without accounting for duration or intensity. Also, being asked daily about their child's participation in organized sports over two weeks might lead parents to feel pressured to answer yes to align with social desirability. Nevertheless, we believe daily reporting is preferred, as a single question at the end of the week could compromise accurate recall. Considering the method of accelerometry, although it is considered a gold-standard measurement of physical activity among preschool aged children in free-living conditions, accelerometers are unable to detect some types of physical activity, e.g., cycling and swimming [26]. Since swimming was the most reported organized sport (6,3%), and is not captured by accelerometers, the real difference in physical activity between those who do and do not participate in organized sports are likely to be larger than our estimates.

## Conclusions

In conclusion, our study underscores a modest positive association between organized sports participation and physical activity among preschool-aged children. Although the association with physical activity is modest in magnitude, it may reflect an early increase that has the potential to persist into adulthood. We also highlight the presence of discrepancies in organized sports participation across parental education and neighborhood socioeconomic index, potentially exacerbating health inequalities. Addressing these socioeconomic disparities requires collaborative efforts from policymakers, educators, healthcare professionals, and community stakeholders to ensure equitable access to organized sports and physical activity opportunities for all children, regardless of their socioeconomic background.

## Supplementary Information

Supplementary Material 1.

## Data Availability

Availability of data and materials The data underlying this article cannot be shared publicly due to Swedish legislation as well as the privacy of individuals that participated in the study. However, by contacting the corresponding author, data code for the analyses can be provided upon reasonable request.

## Abbreviations

CAP: Children’s Physical Activity by Policy
LPA: Light physical activity
MVPA: Moderate to vigorous physical activity
ST: Sedentary time
WHO: The World Health Organization
