# Supplementary material for: Socioeconomic disparities in organized sports participation and physical activity among a population based sample of preschool children: a cross-sectional study
Source: BMC Pediatr. 2025 Apr 16;25:298. doi: 10.1186/s12887-025-05651-3 (PMC12001484; doi:10.1186/s12887-025-05651-3)
Supplement: Supplementary file 1 — Supplementary Material 1. [file 12887_2025_5651_MOESM1_ESM.docx]

**Title**

Socioeconomic Disparities in Organized Sports Participation and Physical Activity Among a Population Based Sample of Preschool Children: A cross-sectional study.

**Authors**

Charlotte Wilén^1^, Viktor H. Ahlqvist^1^, Chen Chu^1,2^, Martin Neovius^3^, Cecilia Magnusson^1,2^, Pontus Henriksson^4^, Micael Dahlén^5^, Erik Sander^1,^ Daniel Berglind^1,2,5^

^1^Department of Global Public Health, Karolinska Institutet, Sweden

^2^Centre for Epidemiology and Community Medicine, Stockholm County Council, Stockholm, Sweden

^3^ Clinical Epidemiology Division, Department of Medicine, Unit, Karolinska Institutet, Sweden

^4^ Department of Health, Medicine and Caring Sciences, Linköping University, Linköping, Sweden

^5^ Center for Wellbeing, Welfare and Happiness**,** Stockholm School of Economics

*Corresponding author*

Daniel Berglind, e-mail: [daniel.berglind@ki.se](mailto:daniel.berglind@ki.se)

**SUPPLEMENTAL MATERIAL**

| **Supplementary Table 1.** Prevalence and Odds Ratios for participation in organized sports across different numbers of occasions, by neighbourhood socioeconomic index and parental education* | | | | | | | | | | | |  |
| --- | --- | --- | --- | --- | --- | --- | --- | --- | --- | --- | --- | --- |
|  |  | **No organized sports** | | |  | **Organized sports >0-1 times a week** | | |  | **Organized sports >1 times a week** | | |
|  |  | **N (%)** | **Prevalence (%) (95% CI)** | **OR (95% CI)** |  | **N (%)** | **Prevalence (%) (95% CI)** | **OR (95% CI)** |  | **N (%)** | **Prevalence (%) (95% CI)** | **OR (95% CI)** |
| **Neighbourhood socioeconomic index** | Very low (Q1) | 386 (23.3%) | 64.4 (60.1-69.1) | 2.1 (1.4-3.1) |  | 105 (13.7%) | 17.5 (14.9-20.7) | 0.9 (0.6-1.2) |  | 108 (21.1%) | 18.0 (14.7-22.2) | ref. |
|  | Low (Q2) | 440 (26.5%) | 60.4 (56.0-65.1) | 2.9 (1.9-4.4) |  | 200 (26.1%) | 27.4 (23.7-31.8) | 2.0 (1.4-2.8) |  | 89 (17.4%) | 12.2 (9.7-15.4) | ref. |
|  | Medium (Q3) | 273 (16.5%) | 54.6 (49.9-59.8) | 2.3 (1.4-3.6) |  | 157 (20.5%) | 31.4 (27.0-36.5) | 2.0 (1.3-3.0) |  | 70 (13.7%) | 14.0 (10.6-18.5) | ref. |
|  | High (Q4) | 321 (19.4%) | 55.7 (49.6-62.6) | 1.7 (1.1-2.7) |  | 148 (19.3%) | 25.7 (21.3-30.9) | 1.2 (0.9-1.7) |  | 107 (20.9%) | 18.6 (15.0-23.0) | ref. |
|  | Very high (Q5) | 238 (14.4%) | 44.8 (38.1-52.7) | ref. |  | 155 (20.3%) | 29.2 (25.0-34.2) | ref. |  | 138 (27.0%) | 26.0 (21.9-30.8) | ref. |
|  |  |  |  |  |  |  |  |  |  |  |  |  |
| **Parental education** | Secondary school | 25 (1.5%) | 67.6 (52.7-86.7) | 1.0 (0.4-2.4) |  | 4 (0.5%) | 10.8 (4.4-26.8) | 0.3 (0.1-0.9) |  | 8 (1.6%) | 21.6 (11.5-40.5) | ref. |
|  | Upper secondary school | 228 (13.8%) | 62.5 (57.4-68.0) | 1.2 (0.9-1.7) |  | 78 (10.2%) | 21.4 (17.1-26.8) | 0.8 (0.6-1.2) |  | 59 (11.5%) | 16.2 (12.7-20.6) | ref. |
|  | University level | 1,212 (73.1%) | 54.1 (51.2-57.1) | ref. |  | 641 (83.8%) | 28.6 (26.6-30.8) | ref. |  | 389 (76.0%) | 17.4 (15.3-19.7) | ref. |
|  | Missing information | 193 (11.6%) |  |  |  | 42 (5.5%) |  |  |  | 56 (10.9%) |  |  |
| *Multinomial logistic regression was used to examine differences in organized sports participation based on parental education and neighbourhood socioeconomic index, with clustering at the preschool level to account for standard error. | | | | | | | | | | | | |

| **Supplementary table 2.** Mean differences in moderate-to-vigorous physical activity, light physical activity, and sedentary time according to main analysis and sensitivity analyses. | | | | | | |
| --- | --- | --- | --- | --- | --- | --- |
| **Sensitivity analysis** | | | | | | |
| **Sedentary time (min)** | | | | | | |
|  | Mean difference (95% CI) | | | | | |
|  | Main analysis** | Without children with height and weight from opposite season* | Without children without measurement from spring* | Without children who participate in swimming** | Without children with no information on parental education** | Without children with < 3 & >16 days of registered organized sports** |
| **Per occasion organized sport** | -3.1 (-5.96,-0.28) | -3 (-5.86,-0.19) | -4.0 (-7.05,-0.89) | -2.7 (-5.75,0.26) | -3.2 (-6.42,0.03) | -3.3 (-6.56,-0.11) |
| **Average number of occasions** |  |  |  |  |  |  |
| **0.0** | ref. | ref. | ref. | ref. | ref. | ref. |
| **>0-1** | -5.3 (-10.37,-0.27) | -4.4 (-9.91,1.06) | -6.0 (-11.33,-0.69) | -4.8 (-9.96,0.44) | -5.6 (-10.88,-0.32) | -5.3 (-10.38,-0.22) |
| **1+** | -9.8 (-16.42,-3.18) | -9.6 (-16.40,-2.88) | -11.6 (-18.72,-4.58) | -10.0 (-17.32,-2.62) | -9.5 (-16.62,-2.37) | -9.7 (-16.41,-2.92) |
| **Light physical activity (min)** | | | | | | |
| **Per occasion organized sport** | -0.4 (-2.38,1.59) | -0.3 (-2.29,1.63) | 0.0 (-2.10,2.01) | -0.5 (-2.62,1.67) | -1.0 (-3.11,1.07) | -0.5 (-2.65,1.57) |
| **Average number of occasions** |  |  |  |  |  |  |
| **0.0** | ref. | ref. | ref. | ref. | ref. | ref. |
| **>0-1** | 1.6 (-2.21,5.32) | 2.1 (-1.79,5.96) | 1.4 (-2.37,5.23) | 0.6 (-3.25,4.53) | 0.9 (-3.04,4.83) | 1.5 (-2.31,5.32) |
| **1+** | 0.1 (-4.42,4.69) | -0.1 (-4.61,4.43) | 1.4 (-3.35,6.15) | 0.7 (-4.12,5.59) | -1.1 (-5.77,3.64) | -0.2 (-4.76,4.26) |
| **Moderate- to vigorous physical activity (min)** | | | | | | |
| **Per occasion organized sport** | 1.1 (0.41,1.79) | 1.0 (0.33,1.72) | 1.4 (0.71,2.11) | 1.1 (0.36,1.89) | 1.4 (0.68,2.13) | 1.1 (0.50,1.78) |
| **Average number of occasions** |  |  |  |  |  |  |
| **0.0** | ref. | ref. | ref. | ref. | ref. | ref. |
| **>0-1** | 2.8 (1.52,4.02) | 2.5 (1.13,3.78) | 2.9 (1.59,4.26) | 2.8 (1.52,4.06) | 2.7 (1.41,4.06) | 2.7 (1.45,3.99) |
| **1+** | 2.8 (1.56,4.06) | 2.7 (1.36,4.00) | 3.4 (2.03,4.69) | 2.9 (1.56,4.26) | 2.9 (1.57,4.20) | 2.7 (1.45,3.90) |
| *Adjusted for age, sex, parental education, accelerometer wear time, obesity status, number of times organized sport is registered, parental education and neighborhood socioeconomic index. | | | | | | |
| **Adjusted for age, sex, parental education, accelerometer wear time, obesity status, number of times organized sport is registered, parental education, neighborhood socioeconomic index, the season in which information on accelerometer data is taken and season of weight/length recording. | | | | | | |
